# Efficacy and safety of nintedanib in patients with advanced idiopathic pulmonary fibrosis

**DOI:** 10.1186/s12890-019-1030-4

**Published:** 2020-01-08

**Authors:** Luca Richeldi, Martin Kolb, Stéphane Jouneau, Wim A. Wuyts, Birgit Schinzel, Susanne Stowasser, Manuel Quaresma, Ganesh Raghu

**Affiliations:** 1 Fondazione Policlinico A. Gemelli IRCCS, Università Cattolica del Sacro Cuore, Rome, Italy; 2 McMaster University and St. Joseph’s Healthcare, Hamilton, Ontario Canada; 3 Hôpital Pontchaillou - CHU de Rennes, IRSET UMR 1085, Université de Rennes 1, Rennes, France; 4 Unit for Interstitial Lung Diseases, Department of Respiratory Medicine, University Hospitals Leuven, Leuven, Belgium; 5 Boehringer Ingelheim International GmbH, Ingelheim, Germany; 6 University of Washington, Seattle, USA

**Keywords:** Clinical trial, Interstitial lung diseases, Tyrosine kinase inhibitor, Vital capacity

## Abstract

**Background:**

The two 52-week INPULSIS trials investigated nintedanib versus placebo in patients with IPF, FVC ≥50% predicted and DLco 30–79% predicted. The 24-week INSTAGE trial investigated nintedanib plus sildenafil versus nintedanib alone in patients with IPF and DLco ≤35% predicted. We used data from INPULSIS and INSTAGE to compare the effects of nintedanib in patients with IPF with less versus more severe impairment in gas exchange at baseline.

**Methods:**

Analyses were conducted in patients treated with nintedanib alone in the INPULSIS and INSTAGE trials and in patients treated with placebo in the INPULSIS trials. Outcomes included the rate of decline in FVC over 24 weeks, the proportions of patients who had a confirmed or suspected idiopathic acute exacerbation over 24 weeks, deaths over 24 weeks, and adverse events. Analyses were descriptive.

**Results:**

In total, 638 and 136 patients received nintedanib alone in the INPULSIS and INSTAGE trials, respectively, and 423 patients received placebo in the INPULSIS trials. Rates of FVC decline were − 52.3 and − 66.7 mL/24 weeks in patients treated with nintedanib alone in INPULSIS and INSTAGE, respectively, and − 102.8 mL/24 weeks in patients treated with placebo in INPULSIS. Confirmed or suspected idiopathic acute exacerbations were reported in 0.6 and 3.7% of patients treated with nintedanib alone in INPULSIS and INSTAGE, respectively, and 2.1% of patients treated with placebo in INPULSIS. Deaths occurred in 2.0, 11.0 and 1.9% of patients in these groups, respectively. Diarrhoea adverse events were reported in 52.5 and 48.5% of patients treated with nintedanib alone in INPULSIS and INSTAGE, respectively, and 16.1% of patients treated with placebo in INPULSIS.

**Conclusions:**

Based on data from the INSTAGE and INPULSIS trials, nintedanib had a similar effect on FVC decline over 24 weeks, and a similar safety and tolerability profile, in patients with IPF and more versus less severe impairment in gas exchange. These data support the use of nintedanib in patients with IPF who have advanced disease.

**Trial registration:**

INPULSIS (NCT01335464 and NCT01335477); INSTAGE (NCT02802345).

## Background

Idiopathic pulmonary fibrosis (IPF) is a progressive fibrosing interstitial lung disease associated with decline in lung function, worsening dyspnoea and quality of life, and considerable mortality [1, 2]. While IPF progresses in all patients, the pattern of disease progression is variable and remains a challenge to predict [3, 4]. A better evidence base for the treatment of severe IPF remains an unmet need [5].

Nintedanib is a treatment for IPF. In the two 52-week INPULSIS trials, nintedanib reduced the annual rate of decline in forced vital capacity (FVC) (mL/year) by approximately 50% compared with placebo in patients with IPF and mild or moderate impairment in lung function at baseline, with an adverse event profile predominantly characterised by gastrointestinal events [6]. In the 24-week INSTAGE trial, nintedanib plus sildenafil was associated with a numerical but not statistically significant benefit on change in St. George’s Respiratory Questionnaire (SGRQ) total score compared with nintedanib alone in patients with IPF and severely impaired gas exchange (diffusing capacity of the lungs for carbon monoxide [DLco] ≤35% predicted) at baseline [7].

Few data are available on the efficacy and safety of antifibrotic therapies in patients with advanced IPF. We used data from the INPULSIS and INSTAGE trials to compare the effects of nintedanib in patients with IPF and less versus more severe impairment in gas exchange at baseline.

## Methods

The designs of the INPULSIS and INSTAGE trials have been published [6, 7]. Briefly, in the INPULSIS trials, patients with FVC ≥50% predicted and DLco 30–79% predicted were randomised 3:2 to receive nintedanib 150 mg twice daily (bid) or placebo for 52 weeks, with a follow-up visit 4 weeks later. The primary endpoint was the annual rate of decline in FVC (mL/year) [6]. In the INSTAGE trial, patients with IPF and DLco ≤35% predicted were randomised 1:1 to receive nintedanib 150 mg bid plus sildenafil or nintedanib 150 mg bid plus placebo for 24 weeks, with a follow-up visit 4 weeks later. The primary endpoint was the change from baseline in SGRQ total score at week 12 [7]. In all these trials, FVC data were converted to per cent predicted values using the European Community for Steel and Coal equations [8]. In the INPULSIS trials, DLco data were converted to % predicted using the equation published by Crapo [9]. In INSTAGE, sites used different equations to calculate per cent predicted values for DLco.

Spirometry was performed at baseline and weeks 2, 4, 6, 12 and 24 in the INPULSIS trials [6], and at baseline and weeks 4, 8, 12, 18 and 24 in the INSTAGE trial [7]. The SGRQ was completed by patients at baseline and weeks 6, 12 and 24 in the INPULSIS trials [6], and at baseline and weeks 4, 12 and 24 in the INSTAGE trial [7]. The SGRQ assesses health-related quality of life (HRQL); there are 50 items and the total score ranges from 0 to 100, with higher scores indicating worse HRQL [10]. In the INPULSIS trials, acute exacerbations were defined as events that met the following criteria: unexplained worsening or development of dyspnoea within 30 days; new diffuse pulmonary infiltrates on chest X-ray and/or HRCT parenchymal abnormalities with no pneumothorax or pleural effusion (new ground-glass opacities) since last visit; causes of the acute worsening, including infection, left heart failure, pulmonary embolism, or any identifiable cause of acute lung injury excluded as per routine clinical practice and microbiological studies [6]. In the INSTAGE trial, an acute exacerbation was defined as an event that met the following criteria: acute worsening or development of dyspnoea, typically of less than 1-month duration; computed tomography with new bilateral ground-glass opacity and/or consolidation superimposed on background pattern consistent with usual interstitial pneumonia pattern; deterioration not fully explained by cardiac failure or fluid overload; extra-parenchymal causes (e.g. pneumothorax, pleural effusion, pulmonary embolism) were excluded [7]. In both the INPULSIS and INSTAGE trials, acute exacerbations were adjudicated by a blinded committee as confirmed, suspected, or not acute exacerbations [6.7]. In the INPULSIS trials, events deemed to be acute exacerbations but did not meet all protocol-specified criteria for an acute exacerbation were classified as suspected acute exacerbations [6]. In the INSTAGE trial, events deemed to be acute exacerbations but had missing CT data were classified as suspected acute exacerbations [7]. In the INSTAGE trial, confirmed/suspected acute exacerbations were further adjudicated as idiopathic or triggered based on the criteria described in the 2016 international working group report [7, 11]. All confirmed/suspected acute exacerbations in the INPULSIS trials were idiopathic [6].

In the current analyses, we investigated changes from baseline in FVC (mL) and SGRQ total score at weeks 12 and 24; the rate of decline in FVC (mL) over 24 weeks; and the proportions of patients who had an absolute decline in FVC ≥5% predicted or died, had an adjudicated confirmed or suspected idiopathic acute exacerbation, or who died from any cause over 24 weeks in patients who received nintedanib alone in the INPULSIS and INSTAGE trials and in patients who received placebo in the INPULSIS trials. For each endpoint, the same statistical approach was used as in the primary analyses [6, 7]. Changes from baseline in FVC and SGRQ total score at weeks 12 and 24 were analysed using mixed effects models for repeated measures. The rate of decline in FVC over 24 weeks was analysed using a random coefficient regression model. These models allowed for missing data, assuming they were missing at random.

Adverse events were reported by the investigators irrespective of causality and coded based on preferred terms in the Medical Dictionary for Regulatory Activities (MedDRA) (version 20.1 in INPULSIS and version 21.0 in INSTAGE) [6, 7]. In the INPULSIS trials, adverse events with onset between the first dose of trial drug and day 195 (or between the first dose and 28 days after the last dose for patients who discontinued trial drug before week 24) were included [6]. In the INSTAGE trial, adverse events with onset between the first dose and up to 28 days after the last dose of trial drug were included. Safety analyses were descriptive [7]. All efficacy and safety analyses were based on patients who received ≥1 dose of trial drug.

## Results

### Patients

Overall, 638 and 136 patients received nintedanib alone in the INPULSIS and INSTAGE trials, respectively; 423 patients received placebo in the INPULSIS trials. In accordance with the eligibility criteria, mean DLco % predicted at baseline was higher in INPULSIS than in INSTAGE (47.2% versus 25.6% predicted). In addition, patients in the INPULSIS trials were younger and had higher FVC % predicted and lower SGRQ total score (indicating better health-related quality of life) than patients in the INSTAGE trial (Table 1).

**Table 1:** Baseline characteristics of patients in the INPULSIS and INSTAGE trials

	INPULSIS		INSTAGE
	Nintedanib (*n* = 638)	Placebo (*n* = 423)	Nintedanib (*n* = 136)
Age, years, mean (SD)	66.6 (8.1)	67.0 (7.9)	70.0 (7.9)
Male, n (%)	507 (79.5)	334 (79.0)	106 (77.9)
Body mass index, kg/m^2^	28.1 (4.6)	27.6 (4.6)	26.5 (4.7)
Race, n (%)			
White	360 (56.4)	248 (58.6)	95 (69.9)
Asian	194 (30.4)	128 (30.3)	39 (28.7)
Other^a^	84 (13.2)	47 (11.1)	2 (1.5)
Time since diagnosis of IPF, years, mean (SD)	1.7 (1.4)	1.6 (1.3)	2.1 (1.8)
FVC, % predicted, mean (SD)	79.7 (17.6)	79.3 (18.2)	66.1 (18.7)
FEV_1_/FVC ratio, %, mean (SD)	81.7 (5.8)	81.7 (6.0)	83.8 (7.6)
DLco, % predicted, mean (SD)^b^	47.4 (13.5)	47.0 (13.4)	25.6 (7.0)
SGRQ total score, mean (SD)^c^	39.5 (19.2)	39.6 (18.5)	54.0 (17.9)

^a^Includes patients with missing data. In INPULSIS, it was not permitted to collect data on race in France. ^b^Corrected for haemoglobin; INPULSIS: *n* = 422 in placebo group; INSTAGE: *n* = 135 in nintedanib group. ^c^INPULSIS: *n* = 624 in nintedanib group and *n* = 419 in placebo group; INSTAGE: *n* = 133 in nintedanib group

### Lung function

**Fig. 1:**
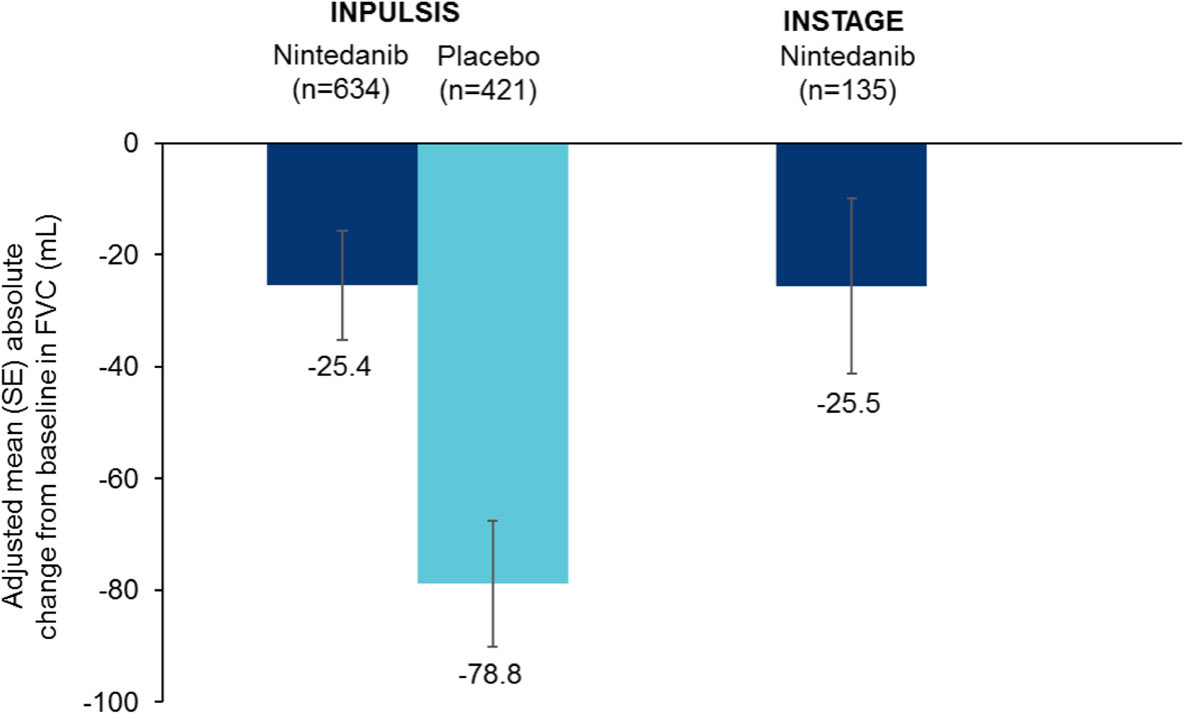
Absolute changes from baseline in FVC (mL) at week 12 in the INPULSIS and INSTAGE trials

**Fig. 2:**
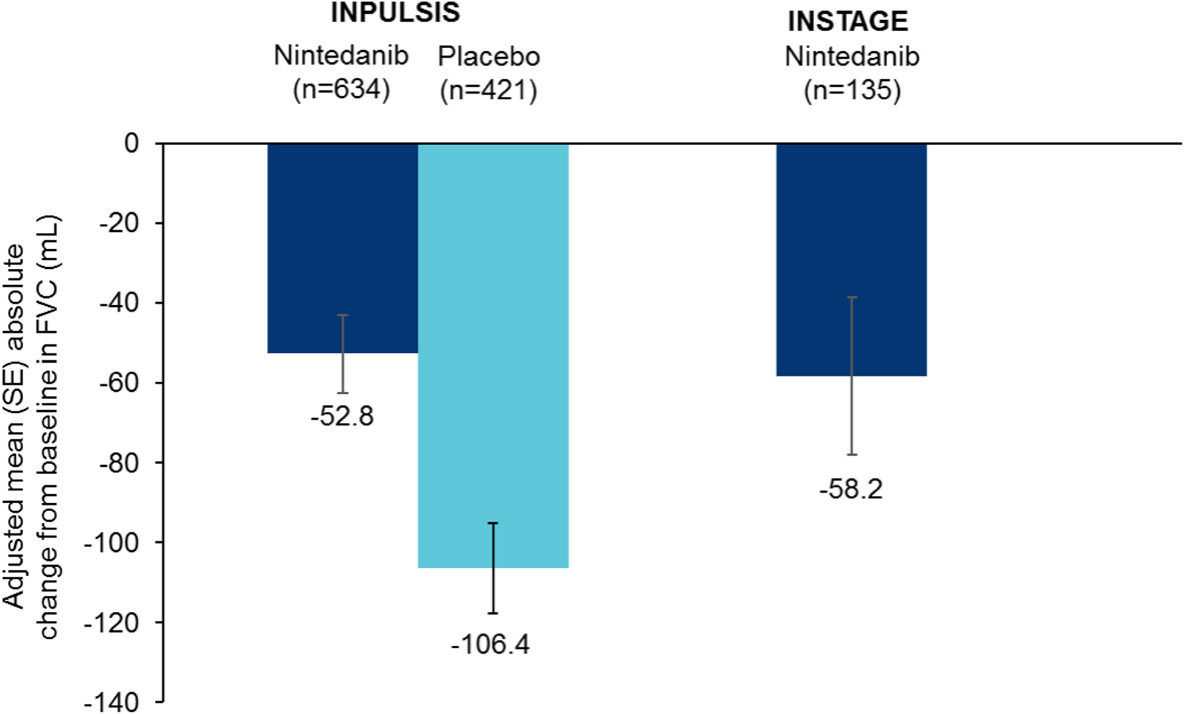
Absolute changes from baseline in FVC (mL) at week 24 in the INPULSIS and INSTAGE trials

**Fig. 3:**
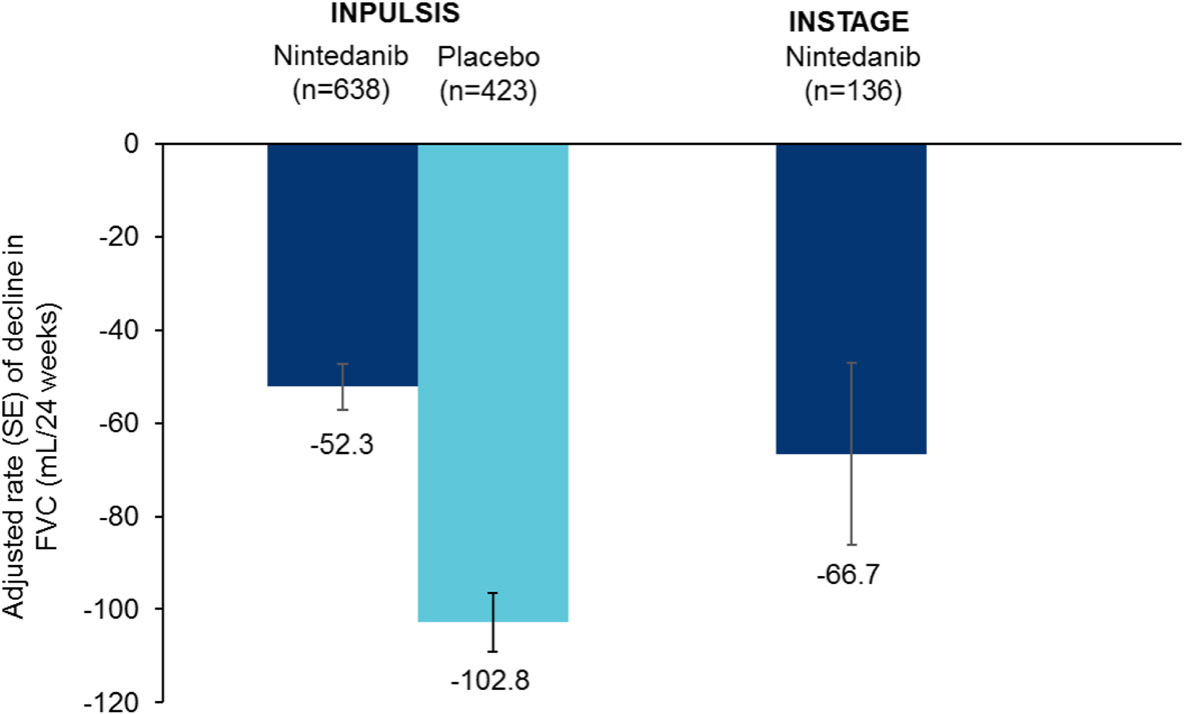
Rate of decline in FVC (mL/24 weeks) in the INPULSIS and INSTAGE trials

Mean (SE) absolute changes from baseline in FVC at week 12 were − 25.4 (9.8) and − 25.5 (15.7) mL in patients treated with nintedanib alone in INPULSIS and INSTAGE, respectively, and − 78.8 (11.3) mL in patients treated with placebo in INPULSIS (Fig. 1). Mean (SE) absolute changes from baseline in FVC at week 24 were − 52.8 (9.8) and − 58.2 (19.6) mL in patients treated with nintedanib alone in INPULSIS and INSTAGE, respectively, and − 106.4 (11.4) mL in patients treated with placebo in INPULSIS (Fig. 2). The rate of decline in FVC (mL) over 24 weeks was consistent with the changes from baseline in FVC (mL) at week 24 (Fig. 3).

Over 24 weeks, an absolute decline in FVC ≥5% predicted or death occurred in 190 (29.8%) and 69 (50.7%) patients treated with nintedanib alone in INPULSIS and INSTAGE, respectively, and 174 (41.1%) patients treated with placebo in INPULSIS.

### Health-related quality of life

Mean (SE) changes from baseline in SGRQ total score at week 12 were 0.38 (0.51) and − 0.77 (1.0) in patients treated with nintedanib alone in INPULSIS and INSTAGE, respectively, and 0.10 (0.61) in patients treated with placebo in INPULSIS. Mean (SE) changes in SGRQ total score at week 24 were 1.05 (0.53) and 2.42 (1.16) in patients treated with nintedanib alone in INPULSIS and INSTAGE, respectively, and 1.78 (0.68) in patients treated with placebo in INPULSIS.

### Acute exacerbations and mortality

Confirmed or suspected idiopathic acute exacerbations occurred in 4 (0.6%) and 5 (3.7%) patients treated with nintedanib alone in INPULSIS and INSTAGE, respectively, and 9 (2.1%) patients treated with placebo in INPULSIS. Deaths occurred in 13 (2.0%) and 15 (11.0%) patients treated with nintedanib alone in INPULSIS and INSTAGE, respectively, and in 8 (1.9%) patients treated with placebo in INPULSIS.

### Safety and tolerability

An overview of adverse events reported in the INPULSIS and INSTAGE trials is provided in Table 2. Adverse events leading to treatment discontinuation were reported in 90 (14.1%) and 23 (16.9%) patients treated with nintedanib alone in INPULSIS and INSTAGE, respectively, and 32 (7.6%) patients who received placebo in INPULSIS. Diarrhoea was reported in 335 (52.5%) and 66 (48.5%) patients treated with nintedanib alone in INPULSIS and INSTAGE, respectively, and 68 (16.1%) patients treated with placebo in INPULSIS, and led to premature treatment discontinuation in 24 (3.8%) and 3 (2.2%) patients treated with nintedanib in INPULSIS and INSTAGE, respectively, and in no patients who received placebo. Serious adverse events were reported in 107 (16.8%) and 44 (32.4%) patients treated with nintedanib alone in INPULSIS and INSTAGE, respectively, and 72 (17.0%) patients treated with placebo in INPULSIS (Table 3).

**Table 2:** Adverse events in the INPULSIS and INSTAGE trials

	INPULSIS		INSTAGE
	Nintedanib (*n* = 638)	Placebo (*n* = 423)	Nintedanib (*n* = 136)
Adverse events	580 (90.9)	345 (81.6)	127 (93.4)
Most frequent adverse events^a^			
Diarrhoea	335 (52.5)	68 (16.1)	66 (48.5)
Nausea	145 (22.7)	25 (5.9)	14 (10.3)
Decreased appetite	53 (8.3)	16 (3.8)	23 (16.9)
Nasopharyngitis	62 (9.7)	43 (10.2)	8 (5.9)
Cough	61 (9.6)	35 (8.3)	13 (9.6)
Vomiting	61 (9.6)	11 (2.6)	10 (7.4)
Dyspnoea	30 (4.7)	25 (5.9)	13 (9.6)
Progression of IPF^b^	33 (5.2)	34 (8.0)	12 (8.8)
Weight decreased	41 (6.4)	8 (1.9)	12 (8.8)
Abdominal pain	53 (8.3)	6 (1.4)	9 (6.6)
Adverse events leading to treatment discontinuation	90 (14.1)	32 (7.6)	26 (19.1)
Most frequent adverse events leading to treatment discontinuation^c^			
Diarrhoea	24 (3.8)	0	3 (2.2)
Nausea	12 (1.9)	0	0
Progression of IPF^b^	7 (1.1)	12 (2.8)	0

Data are n (%) of patients with ≥1 such event. In INPULSIS, events with onset between the first dose of trial drug and day 195 (or between the first dose and 28 days after the last dose for patients who discontinued trial drug before week 24) were included. In INSTAGE, events with onset between the first dose and up to 28 days after the last dose of trial drug were included. ^a^Reported in > 8% of patients in any of the groups shown, based on MedDRA preferred terms. ^b^Corresponds to MedDRA term ‘IPF’, which included disease worsening and acute exacerbations. ^c^Reported in > 1.5% of patients in any of the groups shown, based on MedDRA preferred terms

**Table 3:** Serious adverse events in the INSTAGE and INPULSIS trials

	INPULSIS		INSTAGE
	Nintedanib (*n* = 638)	Placebo (*n* = 423)	Nintedanib (*n* = 136)
Serious adverse events^a^	107 (16.8)	72 (17.0)	44 (32.4)
Most frequent serious adverse events^b^			
Progression of IPF^c^	23 (3.6)	21 (5.0)	9 (6.6)
Pneumonia	14 (2.2)	10 (2.4)	8 (5.9)
Pulmonary hypertension	4 (0.6)	7 (1.7)	4 (2.9)
Respiratory failure	0	0	3 (2.2)
Right ventricular failure	0	0	3 (2.2)
Pulmonary embolism	3 (0.5)	2 (0.5)	2 (1.5)
Lower respiratory tract infection	1 (0.2)	2 (0.5)	2 (1.5)
Respiratory tract infection	0	2 (0.5)	2 (1.5)
Dyspnoea	1 (0.2)	2 (0.5)	2 (1.5)
Acute respiratory failure	2 (0.3)	1 (0.2)	2 (1.5)

Data are n (%) of patients with ≥1 such event. In INPULSIS, events with onset between the first dose of trial drug and day 195 (or between the first dose and 28 days after the last dose for patients who discontinued trial drug before week 24) were included. In INSTAGE, events with onset between the first dose and up to 28 days after the last dose of trial drug were included. ^a^Events that resulted in death, were life-threatening, resulted in hospitalisation or prolonged hospitalisation, resulted in persistent or clinically significant disability or incapacity, were a congenital anomaly or birth defect, or were deemed serious for any other reason. ^b^Reported in ≥1.5% of patients in any of the groups shown, based on MedDRA preferred terms. ^c^Corresponds to MedDRA term ‘IPF’, which included disease worsening and acute exacerbations

## Discussion

Patients with severe lung function impairment have been excluded from most clinical trials of treatments for IPF. As a result, far fewer data are available on the efficacy and safety of therapies in patients with advanced disease than milder disease. Here we have shown, based on data from the INPULSIS and INSTAGE trials, that nintedanib appears to have a similar effect on FVC decline over 24 weeks in patients with IPF irrespective of their severity of gas exchange impairment at baseline. Previous analyses of data from the INPULSIS trials have shown that nintedanib has a consistent effect in reducing FVC decline in subgroups of patients with baseline DLco ≤40% versus > 40% predicted [12] and FVC ≤70% versus > 70% predicted [13]. Data from an interim analysis of INPULSIS-ON, the open-label extension of the INPULSIS trials, suggested that, albeit with the limitations of an open-label design and small sample size, the efficacy and safety of nintedanib were similar in patients with FVC ≤50% predicted at entry into INPULSIS-ON as in patients with less severe disease [14]. A growing body of observational evidence collected in clinical practice suggests that nintedanib is efficacious in reducing disease progression in patients with severe lung function impairment [15–19]. Taken together, these findings support the use of nintedanib in patients with IPF who have advanced disease.

Physicians may be reluctant to treat patients with advanced IPF due to uncertainty about the efficacy and/or safety of antifibrotic therapies in these patients, or to concerns over the tolerability of antifibrotic therapies in patients who are old or have comorbidities [20]. However, many of the patients with IPF who require care in clinical practice have advanced impairment in lung function. Data from 662 patients in the IPF-PRO Registry, a US registry of patients with IPF that was diagnosed or confirmed at the enrolling centre in the past 6 months, showed that 25% of patients had DLco < 31.3% predicted and 25% had FVC < 60.1% predicted [21]. Similar degrees of impairment in FVC and DLco have been observed in other contemporary registries of patients with IPF [22].

Nintedanib had a similar adverse event profile in patients with mild or moderate impairment in gas exchange in the INPULSIS trials and in patients with more severe disease in the INSTAGE trial, albeit with a greater frequency of serious adverse events in patients with more severe disease, as might be expected in a sicker population. Previous analyses of safety data from clinical trials of nintedanib have shown a consistent safety and tolerability profile across trials and patient subgroups [13, 14, 23]. Real-world data from clinical practice suggest that the safety and tolerability profile of nintedanib is similar in patients with IPF who have severe disease as in patients with milder disease, but that patients with more severe disease have a higher rate of treatment discontinuation [16, 18, 24]. This highlights the importance of patient education and proactive management of side-effects in patients prescribed nintedanib for the treatment of IPF [25, 26].

In our analyses, HRQL at baseline, assessed using the SGRQ total score, was worse in patients in the INSTAGE trial than in the INPULSIS trials. Previous studies have also shown that patients with IPF who have more advanced disease based on % predicted values for FVC and/or DLco have worse symptoms, worse exercise capacity, and worse HRQL [27–29]. In both the INPULSIS and INSTAGE trials, changes in SGRQ total score over 24 weeks were small, consistent with previous studies showing that small changes in FVC are not associated with meaningful changes in the patient-reported outcomes commonly used in patients with IPF [30, 31].

As expected, the risk of idiopathic acute exacerbations was higher in patients with more advanced disease in the INSTAGE trial than in patients in the INPULSIS trials. A wealth of data has demonstrated that low FVC and/or low DLco are risk factors for acute exacerbations in patients with IPF [11, 32, 33], possibly because patients with more advanced disease are more vulnerable to insults such as infection that may lead to an acute exacerbation [11]. Similarly, the risk of mortality was higher in patients with more severe impairment in DLco in the INSTAGE trial than in patients in the INPULSIS trials, consistent with previous studies identifying low DLco as a risk factor for mortality in patients with IPF [4, 33, 34].

Our analyses have limitations, including differences between the INPULSIS and INSTAGE trial populations beyond impairment in lung function; the lack of a placebo group in the INSTAGE trial; the short duration of the study; the use of different equations for calculation of per cent predicted values for DLco; and the *post-hoc* nature of the analyses of data from the INPULSIS trials. Nonetheless, our data add to the body of evidence on the efficacy and safety of nintedanib in patients with IPF and advanced disease.

## Conclusion

Based on data from the INPULSIS and INSTAGE trials, nintedanib appears to have a similar effect on FVC decline over 24 weeks, and a similar safety and tolerability profile, in patients with IPF irrespective of their severity of gas exchange impairment at baseline. These data support the use of nintedanib in patients with IPF who have advanced disease.

## Data Availability

All the data generated or analyzed for the purposes of this study are included in the published article.

## Abbreviations

bid: Twice daily
DLco: Diffusing capacity of the lungs for carbon monoxide
FEV_1_: Forced expiratory volume in 1 s
FVC: Forced vital capacity
HRQL: Health-related quality of life
IPF: Idiopathic pulmonary fibrosis
MedDRA: Medical Dictionary for Regulatory Activities
SGRQ: St. George’s Respiratory Questionnaire
